# *Orientia* *tsutsugamushi* Antibodies in Patients with Eschars and Suspected Tickborne Disease

**DOI:** 10.3201/eid3111.250763

**Published:** 2025-11

**Authors:** Haley A. Abernathy, Lauryn Ursery, Brooke A. Merdjane, Dana A. Giandomenico, Ross M. Boyce

**Affiliations:** Author affiliation: University of North Carolina at Chapel Hill, Chapel Hill, North Carolina, USA

**Keywords:** Orientia tsutsugamushi, scrub typhus, chiggers, ticks, Rickettsia, North Carolina, vector-borne infections, bacteria, bacterial infections, tickborne infections, United States

## Abstract

To investigate local transmission of *Orientia tsutsugamushi* by chiggers in North Carolina, USA, we tested remnant serum specimens from patients with eschar undergoing testing for suspected tickborne disease. We identified 11 persons with *O. tsutsugamushi* antibodies, including 4 who were positive by both assays; none had severe clinical manifestations consistent with scrub typhus.

Scrub typhus, caused by *Orientia tsutsugamushi* bacteria and transmitted by Trombiculid mites, affects >1 million persons each year (*1*,*2*). Clinical disease is characterized by fever, headache, and eschars. Untreated, case-fatality rates for the disease can exceed 30% (*2*,*3*).

Most cases of scrub typhus occur in Asia and the Pacific. Although the vectors, commonly known as chiggers, are widely distributed across North America, no autochthonous (i.e., locally acquired) cases have been reported in the United States. In 2023, however, researchers identified *O. tsutsugamushi* in chiggers collected in North Carolina (*4*). To investigate potential transmission to humans in North Carolina, we sought to estimate the seroprevalence of *O. tsutsuguamushi* antibodies and assess the clinical characteristics of persons with evidence of exposure. We hypothesized that cases of eschar are more likely to be caused by endemic tickborne Rickettsiaceae, such as *Rickettsia parkeri* bacteria (*5*).

The protocols of the parent study have been published (*6*). In brief, we collected remanent serum specimens from adult patients undergoing testing for spotted fever group *Rickettsia* or *Ehrlichia* spp. bacteria as part of routine care for acute febrile illness. For the purposes of this study, we selected serum specimens from patients with documented eschar.

We performed serologic testing in parallel by using 2 commercially available kits. We performed all tests according to manufacturers’ instructions and ran the tests with the included positive and negative controls. We first tested samples by using IgM-specific indirect immunofluorescence antibody (IFA) assays against *O. tsutsugamushi* (Fuller Laboratories, https://fullerlaboratories.com) (*7*). We diluted samples to a reciprocal titer of 1:64. In parallel, specimens underwent testing with the Scrub Typhus Detect ELISA-based assays that detect IgM and IgG (InBios International Inc., https://inbios.com). We diluted samples to a reciprocal titer of 1:100. The IgG ELISA kit instructions suggested an optical density (OD) cutoff of 0.37, but the IgM kit did not suggest a cutoff. We applied the 0.37 OD cutoff to the IgG results but also applied several IgM and IgG OD cutoffs from existing literature (*8*,*9*) (Table 1). We estimated seropositivity and used the Clopper-Pearson exact method to calculate 95% CIs.

**Table 1:** Seropositivity estimates based on IgM and IgG ELISA at varying optical density thresholds in study of *Orientia* *tsutsugamushi* bacteria in patients with eschars and suspected tickborne disease, North Carolina, USA, 2020–2022*

Test	Optical density cutoff	Sensitivity	Specificity	Positive, n = 92	% Seropositive (95% CI)
IgG ELISA	0.37	NR	NR	8	8.7 (3.8–16.4)
IgG ELISA (*8*)	1.0	97.5	60	1	1.1 (0.0–5.9)
IgG ELISA (*8*)	1.6	91	75	0	0.0 (0.0–3.9)
IgM ELISA (*8**,**9*)	0.6	96.4	82.7	2	2.2 (0.3–7.6)
IgM ELISA (*8*)	0.492	97.1	79.1	4	4.3 (1.2–10.8)

*NR, not reported.

A total of 138 (5.3%) of 2,593 persons had an eschar documented. Of those, 101 had an adequate sample volume. On the basis of the number of slides and kits available, we selected 87 (86.1%) samples for IFA and 92 (91.1%) samples for ELISA from 83 unique person; some persons had both acute and convalescent samples tested. Of the 83 persons, 35 (42.1%) had illnesses that had been classified as confirmed, probable, or suspected cases of spotted fever group *Rickettsia* (*10*) (Appendix Table 1).

By IFA result, we classified 18 (20.6%) of 87 samples as indeterminate (i.e., weakly positive). By ELISA result, 4 (4.3%) of 92 were positive for *O. tsutsugamushi* IgM and 8 (8.9%) of 90 for IgG; no samples were positive for both. None of the 18 samples that were indeterminate by IFA were positive by ELISA. The 8 samples IgG-positive by ELISA results underwent confirmatory IgG IFA testing at Fuller Laboratories (Appendix Table 2). We observed 50% (4 of 8) agreement in IgG reactivity between ELISA and IFA results at an endpoint titer of 1:128.

**Table 2:** Demographic, clinical, and laboratory test results of persons with antibodies against *Orientia tsutsugamushi* in study of *O.* *tsutsugamushi* among patients with eschars and suspected tickborne disease, North Carolina, USA, 2020–2022*

Person no.	Age, y/sex	IFA result	ELISA result	Tick bite	Fever	Headache	Myalgia	Rash	SFGR classification	*Ehrlichia* classification
1	42/M	IgG+	IgG+	Yes		X		X	Probable	Probable
2	48/M	IgG+	IgG+	Yes	X	X	X		NAC	Unknown
3	66/M	IgG+	IgG+	Yes		X	X	X	NAC	Probable
4	55/F	IgG+	IgG+	Unknown	X	X			NAC	NAC
5	76/F	Negative	IgG+	Yes					Probable	Probable
6	64/M	Negative	IgG+	Yes	X		X		Probable	NAC
7	43/F	Negative	IgG+	Unknown					NAC	Unknown
8	60/F	Unknown	IgM+	Yes			X		NAC	Probable
9	44/M	Negative	IgM+	Unknown					Probable	Unknown
10	78/F	Unknown	IgM+	No	X				Unknown	NAC
11	72/F	Unknown	IgM+	Yes	X		X		Probable	Probable

*IFA, immunofluorescence antibody; NAC, not a case (i.e., did not meet Council of State and Territorial Epidemiologists criteria); SFGR, spotted fever group *Rickettsia*; Unk, unknown or not reported; X, sign or symptom present in patient.

Of the 11 persons with either a reactive IgG or IgM, 7 (63.6%) reported a tick bite. None of the clinical encounters were associated with travel. Fever and myalgia were the most common clinical syndrome (Table 2). All patients were seen in outpatient settings, and none were hospitalized.

Our study identified North Carolina residents with antibodies reactive against *O. tsutsugamushi* bacteria. However, those results must be interpreted cautiously. The presence of antibodies does not indicate active infection but can be a marker of prior exposure. In addition, in a setting where transmission has not been previously reported, positive results are more likely to be falsely positive because of imperfect test performance. However, the presence of antibodies, especially when indicated by both assays, was unexpected and merits investigation.

Including only persons who had reactive antibodies by both assays (n = 4), our estimate of IgG seroprevalence is 4.3% (95% CI 1.2%–10.8%) but, depending on the assay and cutoffs applied, might be as high as 8.9. However, if *O. tsutsugamushi* bacteria were being locally transmitted, we would expect cases of severe illness, which we did not observe.

One limitation of this study is its reliance on serologic results and absence of contextual information, including travel histories and clinical outcomes. In addition, most patients did not have convalescent titers drawn, so we were unable to confirm cases.

We believe the current evidence does not support transmission of virulent *O. tsutsugamushi* strains in North Carolina. Further investigation, ideally by using molecular approaches from clinical samples (e.g., eschar swab samples), is needed. Clinicians should maintain a high level of suspicion for *R. parkeri* rickettsiosis, the seroprevalence of which was nearly 10 times greater than *O. tsutsugamushi*, when evaluating patients with arthropod-associated eschars.

Appendix Additional information about *Orientia* *tsutsugamushi* antibodies in patients with eschars and suspected tickborne disease.
