# Supplementary material for: Orientia tsutsugamushi Antibodies in Patients with Eschars and Suspected Tickborne Disease
Source: Emerg Infect Dis. 2025 Nov;31(11):2187–90. doi: 10.3201/eid3111.250763 (PMC12704536; doi:10.3201/eid3111.250763)
Supplement: Appendix — Additional information about Orientia tsutsugamushi antibodies in patients with eschars and suspected tickborne disease. [file 25-0763-Techapp-s1.pdf]

Article DOI: https://doi.org/10.3201/eid3111.250763
EID cannot ensure accessibility for supplemental materials supplied by authors. Readers who have difficulty accessing supplementary content should contact the authors for assistance.

Orientia tsutsugamushi Antibodies in Patients with Eschars and Suspected Tickborne Disease

Appendix

Appendix Table 1. Laboratory test results and associated case definitions of all persons tested for antibodies against *Orientia tsutsugamushi*

	SFGR IgG			*Ehrlichia* IgG		
Patient	Acute titer result	Conv titer result	Case definition	Acute titer result	Conv titer result	Case definition
1	1:256	–	Probable	1:128	–	Probable
2	1:128	–	Probable	1:512	–	Probable
3	<1:64	–	NAC	–	–	NAC
4	1:256	1:256	Probable	<1:64	–	NAC
5	1:256	1:256	Probable	1:64	–	Suspect
6	1:64	–	Suspect	1:64	–	Suspect
7	<1:64	–	NAC	1:64	<1:64	Suspect
8	<1:64	–	NAC	<1:64	<1:64	NAC
9	<1:64	–	NAC	<1:64	–	NAC
10	<1:64	–	NAC	<1:64	–	NAC
11	<1:64	–	NAC	1:64	–	Suspect
12	1:128	1:64	Probable	1:256	1:128	Probable
13	1:128	<1:64	Probable	1:128	<1:64	Probable
14	<1:64	–	NAC	1:256	–	Probable
15	<1:64	–	NAC	–	–	NAC
16	<1:64	–	NAC	1:256	1:256	Probable
17	1:64	1:64	Suspect	1:64	1:64	Suspect
18	–	–	NAC	1:64	–	Suspect
19	<1:64	<1:64	NAC	1:128	1:128	Probable
20	1:128	–	Probable	–	–	NAC
21	<1:64	–	NAC	1:512	–	Probable
22	1:64	–	Suspect	<1:64	–	NAC
23	<1:64	–	NAC	1:64	–	Suspect
24	<1:64	–	NAC	–	–	NAC
25	1:128	<1:64	Probable	1:512	1:128	Probable
26	<1:64	–	NAC	<1:64	–	NAC
27	1:512	1:128	Probable	1:128	1:64	Probable
28	1:128	–	Probable	<1:64	–	NAC
29	<1:64	–	NAC	–	–	NAC
30	<1:64	–	NAC	<1:64	–	NAC
31	1:1024	–	Probable	<1:64	–	NAC
32	<1:64	<1:64	NAC	<1:64	<1:64	NAC
33	<1:64	–	NAC	<1:64	–	NAC
34	1:512	1:512	Probable	<1:64	–	NAC
35	<1:64	–	NAC	<1:64	–	NAC
36	1:64	–	Suspect	1:64	–	Suspect
37	<1:64	–	NAC	<1:64	–	NAC
38	1:1024	–	Probable	<1:64	–	NAC
39	1:128	1:1024	Confirmed	1:64	<1:64	Suspect
40	1:128	1:64	Probable	<1:64	–	NAC
41	<1:64	–	NAC	1:512	–	Probable
42	1:256	1:1024	Confirmed	<1:64	–	NAC
43	1:64	–	Suspect	<1:64	–	NAC

Patient	SFGR IgG			*Ehrlichia* IgG		
	Acute titer result	Conv titer result	Case definition	Acute titer result	Conv titer result	Case definition
44	1:1024	1:1024	Probable	–	–	NAC
45	<1:64	–	NAC	1:128	–	Probable
46	<1:64	–	NAC	<1:64	–	NAC
47	1:128	–	Probable	1:128	–	Probable
48	<1:64	–	NAC	<1:64	–	NAC
49	1:1024	1:1024	Probable	<1:64	<1:64	NAC
50	<1:64	–	NAC	1:64	–	Suspect
51	<1:64	–	NAC	1:256	–	Probable
52	1:128	1:128	Probable	–	–	NAC
53	<1:64	–	NAC	–	–	NAC
54	1:128	1:64	Probable	<1:64	–	NAC
55	–	–	NAC	<1:64	–	NAC
56	<1:64	–	NAC	1:64	–	Suspect
57	<1:64	–	NAC	<1:64	–	NAC
58	<1:64	–	NAC	1:64	–	Suspect
59	<1:64	–	NAC	1:64	<1:64	Suspect
60	–	–	NAC	<1:64	–	NAC
61	<1:64	–	NAC	–	–	NAC
62	<1:64	–	NAC	<1:64	–	NAC
63	<1:64	–	NAC	<1:64	–	NAC
64	<1:64	–	NAC	<1:64	–	NAC
65	1:1024	–	Probable	1:128	–	Probable
66	1:128	–	Probable	<1:64	–	NAC
67	<1:64	–	NAC	<1:64	–	NAC
68	<1:64	–	NAC	<1:64	–	NAC
69	1:512	–	Probable	<1:64	–	NAC
70	1:128	–	Probable	1:256	–	Probable
71	1:256	1:256	Probable	<1:64	–	NAC
72	1:256	–	Probable	1:256	–	Probable
73	<1:64	–	NAC	–	–	NAC
74	<1:64	–	NAC	<1:64	–	NAC
75	<1:64	–	NAC	1:256	–	Probable
76	<1:64	–	NAC	1:256	–	Probable
77	1:256	–	Probable	1:64	–	Suspect
78	1:512	1:512	Probable	<1:64	–	NAC
79	1:64	1:128	Probable	<1:64	<1:64	NAC
80	<1:64	–	NAC	–	–	NAC
81	<1:64	–	NAC	<1:64	–	NAC
82	<1:64	–	NAC	<1:64	–	NAC
83	<1:64	–	NAC	<1:64	–	NAC

Appendix Table 2. Results for IgG positive specimens at different ELISA cutoffs with comparison to Fuller Laboratories IFA results.

Patient	OD	0.3	0.4	0.6	0.7	1	IFA
1	0.409	+	+	–	–	–	+
2	1.443	+	+	+	+	+	+
3	0.393	+	–	–	–	–	+
4	0.701	+	+	+	+	–	+
5 acute	0.680	+	+	+	–	–	–
5 conv	0.467	+	+	–	–	–	–
6	0.426	+	+	–	–	–	–
7	0.408	+	+	–	–	–	–
